# Inconsistencies in the red blood cell membrane proteome analysis: generation of a database for research and diagnostic applications

**DOI:** 10.1093/database/bav056

**Published:** 2015-06-13

**Authors:** Tamás Hegedűs, Pururawa Mayank Chaubey, György Várady, Edit Szabó, Hajnalka Sarankó, Lia Hofstetter, Bernd Roschitzki, Bruno Stieger, Balázs Sarkadi

**Affiliations:** ^1^MTA-SE Molecular Biophysics Research Group, Hungarian Academy of Sciences, Tűzoltó u. 37-47, H-1094 Budapest, Hungary, ^2^Department of Biophysics and Radiation Biology, Semmelweis University, Tűzoltó u. 37-47, H-1094 Budapest, Hungary, ^3^Department of Clinical Pharmacology and Toxicology, University Hospital Zurich, Raemistrasse 100, CH-8091 Zurich, Switzerland, ^4^Institute of Enzymology, Research Centre for Natural Sciences, Hungarian Academy of Sciences, Magyar tudósok körútja 2, H-1117 Budapest, Hungary and ^5^Functional Genomics Center Zurich, University of Zurich, ETH Zurich, Winterthurerstrasse 190, CH-8057 Zurich, Switzerland

## Abstract

Based on recent results, the determination of the easily accessible red blood cell (RBC) membrane proteins may provide new diagnostic possibilities for assessing mutations, polymorphisms or regulatory alterations in diseases. However, the analysis of the current mass spectrometry-based proteomics datasets and other major databases indicates inconsistencies—the results show large scattering and only a limited overlap for the identified RBC membrane proteins. Here, we applied membrane-specific proteomics studies in human RBC, compared these results with the data in the literature, and generated a comprehensive and expandable database using all available data sources. The integrated web database now refers to proteomic, genetic and medical databases as well, and contains an unexpected large number of validated membrane proteins previously thought to be specific for other tissues and/or related to major human diseases. Since the determination of protein expression in RBC provides a method to indicate pathological alterations, our database should facilitate the development of RBC membrane biomarker platforms and provide a unique resource to aid related further research and diagnostics.

**Database URL:** http://rbcc.hegelab.org

## Introduction

A significant portion (∼30%) of the human proteome consists of membrane proteins and their mutants and polymorphic variants are involved in numerous diseases providing the molecular targets of most of the marketed drugs (1, 2). Due to a multi-step processing and complex regulation of membrane protein expression, in most cases the DNA or mRNA-based information cannot provide appropriate diagnostic information, and only direct protein determinations can serve as proper medical biomarkers. However, tissue sample collection, and the lack of accuracy and sensitivity for quantitative membrane protein detection are the limiting factors in this regard.

A major set of membrane biomarkers is provided by the Cluster of Differentiation (CD) system (see http://www.hcdm.org), mostly applied in haematology and immunology. This set [continuously updated and supervised by the Human Cell Differentiation Markers Workshop (3, 4)] contains now over 500 cell surface markers, including glycoproteins, glycosylated lipids and carbohydrates, all playing important roles in cell function, differentiation and activation. The main advantage of the CD system is that its markers can be detected by validated antibodies and some of them (although still a minority) are routinely used in diagnostic laboratories. Thus, the results provide valuable information of disease states even without knowing the exact identity and function of a given marker. However, a major limitation of this system for membrane proteins is that it includes only those recognized by well-described antibodies, and the quantitation of the CD marker in most cases is inappropriate.

Mass spectrometry-based proteomics may provide both qualitative and quantitative data for membrane protein expression. However, integral membrane proteins are variably recognized by these methods and further difficulties are related to the sampling and quantification of large and hydrophobic membrane proteins (5–8). Since most of the MS approaches do not focus on membrane proteins, they have an inevitable bias towards cytoplasmic proteins. In addition, the need of expensive major equipment hinders their wide-spread diagnostic applications in routine clinical laboratories.

In the case of the human red blood cells (RBCs), the limitations of tissue sample collection and continuous intracellular membrane protein trafficking are not affecting membrane protein determinations. Small blood samples are easy to collect and contain a relatively large number of RBC (about 5 × l0^6^ cells/µl), which have only a single plasma membrane. Several recent studies suggest that uncovering the red cell membrane proteome may provide the basis of valuable diagnostic tools (9–11). However, even highly purified RBC samples may contain immature or contaminating cells, and minor contaminants may have a major impact on the results of proteomics. Thus, a vigorous control is necessary in this regard.

There have been numerous studies using different approaches (e.g. MS, 2D electrophoresis and antibody detection) attempting to describe the RBC membrane proteome (8, 11). CD markers for the RBC are available, and the blood group systems are also provided in major databases. Surprisingly, we found major inconsistencies in these data, thus their medical diagnostic use is hampered by the contrasting findings, the ambiguous protein names, outdated database identifiers and the variable format of data. Mining information on RBC proteins from laborious reviews merging data from different studies (6, 10) is still extremely difficult, and currently there is no available central and searchable database for the membrane proteins expressed in RBCs.

In this study, we aimed to generate a tool for the personalized medical diagnosis of diseases associated with membrane proteins, by establishing an integrated platform of the RBC membrane proteome. First, we performed MS studies on human RBC membrane preparations which were further washed in alkaline milieu to remove cytoplasmic and extracellular adhering proteins. Later, we compared our dataset with other MS-based, CD cluster-defined and blood group-specific databases. Based on these results, we have generated an integrated database based on various RBC-relevant sources, for a research and diagnostic biomarker application of the RBC membrane proteome.

## Methods

### MS analysis of red cell membrane proteins

#### RBC ghost preparation

Membrane was prepared from 50 ml of blood provided by healthy donors with a written consent in a project with approved ethical committee permission. This study was approved by the regional ethical committees (Department of Health, Office of Hungarian Government, Budapest, Hungary), and all procedures were performed in accordance with the Declaration of Helsinki. RBC was isolated and RBC membranes (ghosts) were prepared according to the original methods described by Schatzmann and Rossi (12) and Wolf (13). These processes involved the removal of contaminating cells, including platelets and white-blood cells (confirmed by flow cytometry examinations), during several washes of the red blood cells. In the flow cytometry measurements, RBCs were labelled with WGA-Alexa488, while Draq5 nuclear stain was used to visualize all white blood cells. For selectively labelling the platelets, an FITC-conjugated anti-CD61 antibody was applied. We found that after three washes of the blood samples, the RBC/total WBC ratio decreased from an original mean value of 0.27 to 0.0002%, while the RBC/platelet ratio decreased from a mean of 2.47 to 0.011%. This means the presence of about 2 WBC/10^6^ RBC, and about 1 platelet/10^4^ RBC in the final RBC preparation. Potential reticulocyte contamination was also examined by Giemsa staining of the RBC preparation, and reticulocyte count after three washes (removing the upper layer of RBCs) decreased from 2 to <0.02%. In order to remove any membrane-associated cytoplasmic or plasma proteins from ghosts, the white RBC membranes (5 mg protein/ml) were diluted with 20-fold volume of 0.5 mM Tris/HCl, 0.05 mM DTT (pH 8.5), incubated for 30 min at 4°C and for 15 min at 37°C, then homogenized with a 27-gauge needle. The membranes were further washed first with 10 mM Tris/HCl, 0.5 mM EDTA, pH 8.0 and then twice with large volumes of 10 mM Tris/HCl, pH 7.4. The membranes were resuspended at 2 mg protein/ml in 140 mM KCl and 20 mM Tris/HCl, pH 7.4 and kept frozen until further use.

#### Membrane protein extraction

Erythrocyte membrane (ghost) extraction with carbonate to remove adhering proteins was performed according to Fujiki *et al.* (14) in the presence of Mini complete protease inhibitor (Roche Applied Biosystems, Switzerland) for 1 h at 4°C. Extracted membranes were collected after ultracentrifugation for 1 h at 100 000g_av_. The supernatant was discarded and the pellet was resuspended in 200 µl sucrose (250 mM) using a syringe with a 25-gauge needle. The protein estimation was carried out using BCA protein assay kit (Interchim, France).

#### In-solution digestion

200 µg of protein was first deglycosylated with PNGase F (NEB) at 37°C [overnight at 500 rpm, at RT (15)]. The sample was diluted with 20 mM ammonium bicarbonate (pH 8) and 0.1% Rapigest SF surfactant (Waters, USA). The proteins were reduced using 5 mM DTT (30 min at 60°C, 700 rpm) followed by alkylation with 45 mM iodoacetamide (10 min at RT in the dark). The reaction was quenched by adding 30 mM DTT (10 min at RT). The resulting sample was first digested using trypsin (sequencing grade; Promega, Switzerland) at a ratio of 1:20 (3.5 h at 37°C at 700 rpm) followed by centrifugation for 10 min, 13 000 g) and supernatant (S1) was collected. The pellet was further digested with chymotrypsin and trypsin at a ratio of 1:1 (overnight at 37°C at 700 rpm). The reaction was stopped by adding 50% acetonitrile and 0.1% trifluroacetic acid (37°C, 30 min at 700 rpm) and the supernatant was collected (S2). S1 and S2 were pooled for further analysis.

#### Pre-fraction by Strong Cation Exchange (SCX)

Peptides obtained above were vacuum dried, resuspended in buffer A (10 mm KH_2_PO_4_, pH 2.7, in 25% acetonitrile), and loaded onto a 2.1 × 200 mm polysulfoethyl aspartamide A column (PolyLC, USA) on an Agilent HP1100 binary HPLC system (Agilent Technologies, USA). Peptides were eluted with an increasing KCl gradient (10–40 min, 0–30% buffer B; 40–60 min 30–100% buffer B; 10 mM KH_2_PO_4_, pH 2.7 and 500 mM KCl in 25% acetonitrile) and fractions of ∼0.6 ml collected. Eluted peptides were pooled into 17 fractions based on the chromatogram, and desalted using Ziptips (Millipore, Switzerland) following the vendors protocol.

#### MS analysis

Desalted samples were vacuum concentrated and resuspended in 3% acetonitrile and 0.1% formic acid, before injecting on LTQ Orbitrap XL mass spectrometer (Thermo Fischer Scientific, Germany) coupled to an Eksigent nano LC system (Eksigent Technologies, USA). Peptide separation was made using self-packed (75 µm × 80 mm) reverse phase column packed with C18 material (AQ 3 µm, 200 A; Bischoff GmbH, Germany) as previously described in Mayank *et al.* (16).

#### Data refinement

**Figure 1.**
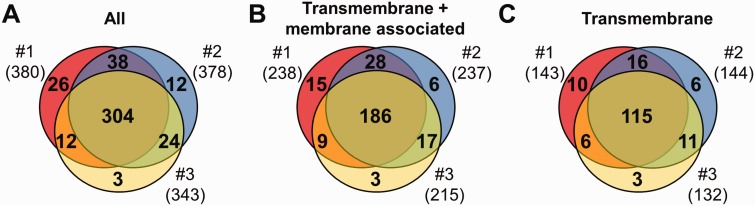
MS results of 3 independent red cell membrane samples. (**A**) These MS experiments were reproducible, as indicated by the high number (304) of proteins identified in three individual experiments. (**B, C**) Detection of the RBC transmembrane and membrane-associated proteins was also efficient. For methodological details see ‘Methods’ section.

After data collection peak lists were generated using Mascot Distiller 2.3 (Matrix Science Inc., UK) and searched against a human database from SwissProt (release December 2012) concatenated with its decoyed version and an in-house build contaminant database using the Mascot search algorithm (Mascot 2.3; Matrix Science Inc., UK). The following search parameters were used: precursor ion mass tolerance 10 ppm; fragment ion mass tolerance 0.8 Da; trypsin digestion (one missed cleavage allowed); fixed modifications of carbamidomethyl of cysteine; variable modification oxidation of methionine as well as pyro-Glu formation of peptide N-term glutamine. After the Mascot search data were further evaluated using Scaffold 4.3 (Proteome Software Inc., OR, USA). Thresholds for protein identification were set to 95% protein probability; 99% peptide probability and two peptides per protein. Final data processing was carried out using Microsoft Excel 2010 (Microsoft Corp) and presented in the Figure 1 and Supplementary Table S1.

### Database development and analysis

#### Semi-automatic data mining

To identify a protein from different data sources, gene names and identifiers (e.g. IPI, UniProtKB, etc.) were queried in different ways. In the case of publications, the pdf file of the supplementary tables was converted to html format, while in the case of a web resource (e.g. hRBCD, BGMUT) contents were downloaded in html or text format. Python scripts were written and used to map gene names and identifiers to unique, when possible reviewed, UniProtKB entries. When this process failed, we tried to assign gene names or database identifiers manually to UniProtKB records. For example, in the case of unmatched IPI (International Protein Index) identifiers, different versions of the retired IPI database were downloaded (http://www.ebi.ac.uk/IPI) and searched. To convert GI numbers to UniProt accessions, the mapping API of UniProtKB was employed. In certain, relatively low number of cases our efforts to identify the target protein unambiguously failed because of obsolete entries and retired databases that are not existing or maintained any more.

#### Software and tools

Membrane and membrane associated proteins were visualized using various online resources: TMHMM2.0 (1), Protter (17) (http://wlab.ethz.ch/protter/start/), data from The Human Transmembrane Proteome Database (http://htp.enzim.hu), and different sections of UniProtKB including ‘Subcellular location’ and ‘Features’ were considered. The database uses the *MySQL* relational database back-end (http://www.mysql.com) for data storage. For data access, the *SQLAlchemy* (http://www.sqlalchemy.org) object-relational mapper libraries were used. The web interface was created based on the *TurboGears* web framework (http://turbogears.org) and the *Genshi* templating library (http://genshi.edgewall.org). Selected data fields of UniProtKB (18) presented in the entry page of a protein are stored in a highly organized relational way, while the whole UniProtKB records are stored in a separate table, used exclusively for full text searches. Full text data from the sources were also inserted for certain proteins when it was possible [e.g. in the case of BGMUT (19)]. To aid searches for proteins connected to diseases, we included OMIM (Online Mendelian Inheritance in Man, OMIM®. McKusick-Nathans Institute of Genetic Medicine, Johns Hopkins University, Baltimore, MD, http://omim.org) data, mapped to protein entries for full text query possibility. Information about isoforms identified in our MS experiments is also inserted and made searchable. Data analysis was performed using python scripts and the R statistical package.

## Results and discussion

### MS analysis of human RBC membranes and database comparisons

When searching for the presence of red cell membrane proteins in MS datasets on RBC, significant differences in the identified proteins between various MS studies were observed (see below). In order to test for the presence of membrane proteins and to understand the possible sources of differences, an MS-based proteomics study on isolated membranes (ghosts) of human RBCs was performed in house. The preparation method (see ‘Methods’ section) was selected based on previous experience that this approach removes contaminating cell types and loosely membrane-bound cytoplasmic or plasma proteins most efficiently. In order to assess the quality of our MS data, a comparative analysis with published human peripheral blood constituents proteome (20) with a particular emphasis on proteins known to be expressed in potentially contaminating membranes. This analysis revealed that most of the identified proteins in our study are not present in blood constituents other than erythrocytes (Supplementary Figure S1; Table 2) and that the level of contamination with non-RBC markers was minimal (21). As an important technical point, in order to try to enhance the efficacy of proteolysis in our MS studies, we included extensive deglycosylation of the proteins and screened for the most optimal proteolysis conditions (22).

Three independent experiments were performed to detect membrane proteins, and the data are presented in Figure 1. We have identified altogether 419 proteins, 264 of them had predicted transmembrane domains or were labelled as membrane-associated proteins in SwissProt (Figure 1A and B). These 264 ‘membrane’ proteins found in our MS study could be classified as integral, transmembrane (TM) proteins (167), and membrane-associated (97) proteins. The other identified proteins in our RBC membrane preparations are probably soluble proteins, variably attaching to the membrane (155). Regarding the transmembrane proteins, in the three preparations we found 143–144–132 TM proteins, from which 115 were found in all three membrane preparations (Figure 1C). The small differences of the samples may result from both technical reasons and differences in protein expression of individual blood donors.

**Figure 2.**
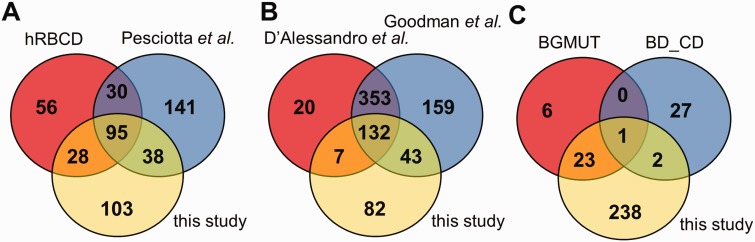
Comparison of the coverage of RBC transmembrane and membrane-associated proteins in different datasets. (**A**) The presence of membrane proteins in MS datasets, (**B**) in comprehensive reviews and (**C**) in highly validated data sources are compared with membrane proteins identified in our MS study. hRBCD, human RBC Database; Pesciotta *et al.*, D’Alessandro *et al.*, and Goodman *et al.* are references to (6, 10, 11); BGMUT and BD_CD mark the human blood group database and the CD marker table provided by BD, respectively.

In the following, we have made a detailed comparison of our MS study results with various available data sources. The largest and most complete datasets of the erythrocyte membrane proteins can be found in MS-based studies. However, when we analysed several of the most comprehensive MS-based datasets (6, 7, 10), we found only scattered overlaps (Figure 2).

Interestingly, out of the 264 membrane proteins identified in our current MS experiments, a relatively high number of proteins (141) were not listed in hRBCD, and 131 were not found in the dataset of Pesciotta *et al.* (11) In contrast, our study could not detect 86 proteins out of the 209 membrane proteins listed in hRBCD and 171 out of the 304 proteins listed by Pesciotta *et al* (11) (Figure 2A).

Our analysis of these and other MS-based data from publications (21, 23) was rendered to be more difficult or impossible in certain cases. An incomplete list of challenges includes identifiers pointing to non-human (e.g. rabbit, dog, mouse) protein entries, different GI numbers listed but pointing to the same gene, some of the GI numbers listed which do not match the corresponding UniProtKB accession listed in the same Supplementary table, etc. Therefore we turned to reviews that already processed various MS-based studies. A major MS-based list of red cell membrane proteins has been compiled by D’Alessandro *et al.* (6) Although this list contains 512 membrane proteins, 125 proteins found in our study are not present in this dataset (Figure 2B). The recent RBC protein collection by Goodman *et al.* (10) lists 687 potential membrane proteins, with 85 unique entries, still leaving out a high number of RBC membrane proteins found in other sources we investigated (Figure 2B).

The RBC membrane proteins included in the CD system for erythrocyte antigens (http://www.hcdm.org), and proteins providing the molecular basis of blood group antigens, compiled in the human blood group database (BGMUT) at the NCBI web site (19) (http://www.ncbi.nlm.nih.gov/projects/gv/rbc/xslcgi.fcgi?cmd=bgmut/home) provide additional data sources in this regard. In both cases cell surface expressed proteins are validated by antibodies, and in many cases the related genetic background, modification patterns, etc. are also provided. Again, in a comparative analysis, we found that the CD list (containing 30 RBC membrane proteins) has 25 unique entries relative to all other databases, and the BGMUT (containing 30 red cell membrane proteins) has three unique entries. Our MS-based dataset does not contain 27 members from the CD list and six proteins from BGMUT (Figure 2C; Supplementary Tables S3 and S4). Only further detailed experiments by applying various methodologies may help to resolve these inconsistencies. However, a properly constructed database may significantly help to explore these questions and promote further research in this regard.

### Generation of RBCC, an interactive database for red cell membrane proteins

The use of the currently available resources is cumbersome or not sufficient to identify a membrane protein in RBC as a potential biomarker. Bioinformatics studies suffer from identifying all RBC proteins from MS data sources (24). To overcome these difficulties we have generated the RBCC (Red Blood Cell Collection), a database and a web application allowing the storing and accessing of all experimentally identified RBCs proteins focusing the RBC membrane proteins (http://rbcc.hegelab.org).

In this database, we have integrated knowledge from various resources, including the hRBCD (7), reviews (6, 10), the BGMUT database of blood group system (19) and the CD marker table provided by BD. Gene names and preferentially UniProtKB IDs were identified either automatically or manually. UniProtKB IDs are used in our database to uniquely identify proteins, since UniProtKB is currently one of the most stable and reliable protein identification systems (18). Moreover, our data are UniProtKB centred in a way that the basic properties of proteins (e.g. name, function, genetic variants, cross-references, etc.) are taken from this database, since it is a manually curated with high reliability.

The resulting RBCC database now contains 2638 unique protein entries, from which 846 are presumed ‘membrane’ proteins (either labelled as transmembrane or membrane-associated proteins), and out of these 376 are labelled as TM proteins, that contain predicted transmembrane regions. By default, the search function is currently constrained to the ‘membrane’ proteins in the database.

One of the main purposes of a database design is to help users with the ease of data accessibility. While it is usually legitimate and necessary to create the data storage in a relational database management system (RDBMS), this implementation does not allow an easy, Google-like search option—a problem faced on most of the biological databases available on the Internet. To have an improved and user-friendly search option (Supplementary Figure S2), we have employed the full text search possibilities of the RDBMS. In the result set and in the entry page of a protein (Supplementary Figure S3) selected data fields of UniProtKB are presented.

Since in many cases the data sought by the users may not be present in UniProtKB, records from other databases are also inserted into the RBCC database. As an example, in order to help to find RBC membrane protein biomarkers related to diseases, the OMIM (http://omim.org) records for the identified RBC membrane proteins are also presented. In addition, in our database registered users can make important comments (e.g. on quality of antibodies; verification of the presence of a protein in RBC by biochemical studies) which are also visible for following users.

In order to assess the genetic background of a selected RBC membrane protein as a potential biomarker, our web application lists the available genetic variants and also hyperlinks to cross-references selected from UniProtKB. In addition, we provide a link to Antibodypedia (25), containing validated antibodies against proteins, that readily aid the selection of an antibody to be tested in immunoedetection, e.g. by flow cytometry. Antibodypedia is an alternative of the CD antibody panels, containing also validated antibodies for the human proteome, not exclusively for the few hundred proteins in the CD system.

### Comparative analysis of data for membrane proteins in RBCs

Recent databases provided a surprisingly large number of previously unexplored membrane proteins in the single plasma membrane compartment of the human erythrocytes. Moreover, it has been suggested that the quantitative expression levels of certain membrane proteins in the easily accessible RBC are closely related to different disease conditions (9, 26). However, when trying to analyse and compare the available databases, we found an unexpected confusion and lack of confidence for RBC membrane proteins searches.

Therefore, we have performed an own MS study on the isolated human RBC, and compared it with different available resources before assembling into a database for RBC membrane proteins (http://rbcc.hegelab.org). As detailed above, the current database containing 846 ‘membrane protein’ entries, based on many of the above-mentioned datasets, most probably represents an overestimation of the potential RBC membrane protein constituent. Therefore, we have also introduced ‘confidence’ levels to evaluate the potential validity of the listed proteins. A low level was set, if the protein was identified only semi-automatically from reviews, in which case we are not able to assess the quality of the large number of sources in these review papers. A medium level was specified when the protein is present in either hRBCD (7), in the study of Pesciotta *et al.* (11), or in our MS samples. We compared the methodologies and the results of these MS studies and drew the conclusion that the differences may arise from problems of membrane protein MS (e.g. variability in protease accessibility or sample components caused by hydrophobic properties resulting in aggregation and/or adhesion to tube walls). We defined a high confidence level, when the protein was present in at least two MS studies [hRBCD (7), PM22954596/Pesciotta *et al.* (11), and our work] or is an established blood group or CD marker. Although this is only a temporary, coarse-grained setting of confidence levels in the database, this approach and further related studies may significantly help the decisions for selecting RBC membrane proteins as possible targets for research or diagnostic markers.

In order to allow assessing whether or not a protein is a candidate diagnostic biomarker, we have extended the RBCC database to cover the available data for membrane protein function, the variability of the genetic background as well as the relationship with genetically determined human diseases. Thus, the RBCC protein database and its search and command options presented here may significantly help the current, accelerated development of new biomarkers helping both stratified/personalized diagnostics and therapy.

This database now contains an unexpected large number of validated membrane proteins previously thought to be specific for selected tissues and calls attention to RBC proteins related to major human diseases. As examples, among the RBC membrane proteins we find key ABC transporters (e.g. ABCC1-MRP1, ABCC4-MRP4, ABCG2-BCRP), more than 20 different solute carrier (SLC) type transporters, and an important copper-transporting ATPase. Virus and other infectious agent receptors, proteins involved in membrane lipid organization and modulation (e.g. phospholipid transporters and scramblases), signal transduction-related proteins (e.g. interleukin receptors, RAS proteins, LIF receptor, G-protein regulated protein kinases, PI and PIP kinases, RAB, RAC, RAP and RAS proteins), membrane scaffolding, trafficking and cell adhesion proteins (e.g. flotillin 1 and 2, syntaxins, basigin, VCAM, ICAM) are also present with ‘high confidence’.

Many of the proteins identified in red cell membrane are involved in the development of either monogenic or polygenic diseases, e.g. proteins related to Alzheimer disease (e.g. the amyloid beta protein, alpha-synuclein, amyloid beta precursor protein-binding protein 1, clusterin, presenilin-1; nicastrin, acetylcholinesterase, basigin, protein disulfide-isomerase), to metabolic syndromes (e.g. GLUT1, GLUT3, GLUT4, urea transporters, ferroportin, ABCG2, monocarboxylate transporter, aquaporins, cAMP-dependent protein kinase, G-protein subunits, diacylglycerol kinase, RHO kinases, nicastrin), or to hypertension (e.g. glucose transporters, the Band3 anion transporter, the K-Cl co-transporter 3, the KCNN4-Gardos channel, aquaporin1, plasma membrane ATPases, adducins, ankyrin, Hras, Kras and Nras, G alpha protein, IL receptors, dynamin, interferon and TNF receptors). A follow-up study exploring the quantitative expression of the RBC membrane proteins, as already performed for ABCG2 (26), may provide key information regarding some of the above-listed candidates.

A reference RBC proteome and known caveats in determining the RBC proteome would be important also for therapeutic developments, involving erythrocytes generated from induced pluripotent stem cells (iPSC) (27). Although comparison of iPSC-derived erythroid cells to erythrocytes indicated highly overlapping proteomes, a significant number of both soluble and membrane proteins (e.g. CD44 responsible for the Indian blood group system, PMCA4, ABCG2) were not identified in cell lines in this MS-based study. These difficulties could lead to misinterpretation of the similarity levels (proteins present in the reference cell line but not detected and absent in the iPSC-derived erythroid cells), thus may result in false directions in therapeutic developments.

It is interesting to note that when performing the analysis of our extended database, we did not find several clinically important membrane proteins, indicated by numerous earlier studies to be present in the erythrocyte membrane. There are several examples of such ‘outliers’, including the insulin receptor, the beta adrenergic receptor and several ABC and SLC transporters. This may be a hint that even large-scale proteomic studies may miss important information and further results of biochemical, genetic and immunological studies should be combined to have a complete knowledge in this regard. In order to compensate for this clearly incomplete feature of the current RBC databases, we provided an ‘open correctibility’ function in this database, so that the content of the database can be updated based on comments from registered users.

## Conclusions

Numerous human membrane proteins became accepted clinical biomarkers and the determination of the easily accessible RBC membrane proteins may provide new diagnostic possibilities in this regard. Since the current databases including our membrane specific proteomics presented in this study show large variations, we generated a comprehensive and expandable database for the RBC membrane proteins. The integrated web database now refers to proteomic, genetic and medical databases as well, and contains an unexpected large number of validated RBC membrane proteins previously thought to be specific for other tissues and/or related to major human diseases. This study should facilitate the development of RBC membrane biomarker platforms and provide a unique resource to aid further research and diagnostics.

## Supplementary Data

Supplementary data are available at *Database* Online.

Supplementary Data

## References

[1] Krogh A. Larsson B. von Heijne G. (2001) Predicting transmembrane protein topology with a hidden Markov model: application to complete genomes. J. Mol. Biol., 305, 567–580. 11152613 10.1006/jmbi.2000.4315

[2] Overington J.P. Al-Lazikani B. Hopkins A.L. (2006) How many drug targets are there? *Nat*. Rev. Drug Discov., 5, 993–996. 10.1038/nrd2199 17139284

[3] Zola H. Swart B. (2005) The human leucocyte differentiation antigens (HLDA) workshops: the evolving role of antibodies in research, diagnosis and therapy. Cell Res., 15, 691–694. 16212875 10.1038/sj.cr.7290338

[4] Zola H. Swart B. Nicholson I. (2005) CD molecules 2005: human cell differentiation molecules. Blood, 106, 3123–3126. 16020511 10.1182/blood-2005-03-1338

[5] Alexandre B.M. (2010) Proteomic mining of the red blood cell: focus on the membrane proteome. Expert Rev. Proteomics, 7, 165–168. 20377381 10.1586/epr.09.96

[6] D’Alessandro A. Righetti P.G. Zolla L. (2010) The red blood cell proteome and interactome: an update. J. Proteome Res., 9, 144–163. 19886704 10.1021/pr900831f

[7] Pasini E.M. Kirkegaard M. Mortensen P. (2006) In-depth analysis of the membrane and cytosolic proteome of red blood cells. Blood, 108, 791–801. 16861337 10.1182/blood-2005-11-007799

[8] Krishnan S. Gaspari M. Della Corte A. (2011) OFFgel-based multidimensional LC-MS/MS approach to the cataloguing of the human platelet proteome for an interactomic profile. Electrophoresis, 32, 686–695. 21337587 10.1002/elps.201000592

[9] Varady G. Cserepes J. Nemeth A. (2013) Cell surface membrane proteins as personalized biomarkers: where we stand and where we are headed. Biomark. Med., 7, 803–819. 24044572 10.2217/bmm.13.90

[10] Goodman S.R. Daescu O. Kakhniashvili D.G. (2013) The proteomics and interactomics of human erythrocytes. Exp. Biol. Med. (Maywood), 238, 509–518. 23856902 10.1177/1535370213488474

[11] Pesciotta E.N. Sriswasdi S. Tang H.Y. (2012) A label-free proteome analysis strategy for identifying quantitative changes in erythrocyte membranes induced by red cell disorders. J. Proteomics, 76, 194–202. 22954596 10.1016/j.jprot.2012.08.010 PMC3508302

[12] Schatzmann H.J. Rossi G.L. (1971) (Ca^2+^+ Mg^2+^)-activated membrane ATPases in human red cells and their possible relations to cation transport. Biochimica et Biophysica Acta, 241, 379–392. 4258479 10.1016/0005-2736(71)90037-x

[13] Wolf H.U. (1972) Studies on a Ca^2+^-dependent ATPase of human erythrocyte membranes. Effects of Ca^2+^ and H+. Biochimica et Biophysica Acta, 266, 361–375. 4261006 10.1016/0005-2736(72)90094-6

[14] Fujiki Y. Hubbard A.L. Fowler S. (1982) Isolation of intracellular membranes by means of sodium carbonate treatment: application to endoplasmic reticulum. J. Cell Biol., 93, 97–102. 7068762 10.1083/jcb.93.1.97 PMC2112113

[15] Kita Y. Miura Y. Furukawa J. (2007) Quantitative glycomics of human whole serum glycoproteins based on the standardized protocol for liberating N-glycans. Mol. Cell. Proteomics MCP, 6, 1437–1445. 10.1074/mcp.T600063-MCP200 17522412

[16] Mayank P. Grossman J. Wuest S. (2012) Characterization of the phosphoproteome of mature Arabidopsis pollen. Plant J., 72, 89–101. 22631563 10.1111/j.1365-313X.2012.05061.x

[17] Omasits U. Ahrens C.H. Muller S. (2014) Protter: interactive protein feature visualization and integration with experimental proteomic data. Bioinformatics, 30, 884–886. 24162465 10.1093/bioinformatics/btt607

[18] Arnold C.D. Gerlach D. Stelzer C. (2013) Genome-wide quantitative enhancer activity maps identified by STARR-seq. Science, 339, 1074–1077. 23328393 10.1126/science.1232542

[19] Patnaik S.K. Helmberg W. Blumenfeld O.O. (2012) BGMUT: NCBI dbRBC database of allelic variations of genes encoding antigens of blood group systems. Nucleic Acids Res., 40, D1023–D1029. 22084196 10.1093/nar/gkr958 PMC3245102

[20] Haudek V.J. Slany A. Gundacker N.C. (2009) Proteome maps of the main human peripheral blood constituents. J. Proteome Res., 8, 3834–3843. 19580323 10.1021/pr801085g

[21] Bosman G.J. Lasonder E. Groenen-Dopp Y.A. (2012) The proteome of erythrocyte-derived microparticles from plasma: new clues for erythrocyte aging and vesiculation. J. Proteomics, 76, 203–210. 22669077 10.1016/j.jprot.2012.05.031

[22] Gupta N. Wollscheid B. Watts J.D. (2006) Quantitative proteomic analysis of B cell lipid rafts reveals that ezrin regulates antigen receptor-mediated lipid raft dynamics. Nat. Immunol., 7, 625–633. 16648854 10.1038/ni1337

[23] van Gestel R.A. van Solinge W.W. van der Toorn H.W. (2010) Quantitative erythrocyte membrane proteome analysis with Blue-native/SDS PAGE. J. Proteomics, 73, 456–465. 19778645 10.1016/j.jprot.2009.08.010

[24] Szczesny P. Mykowiecka A. Pawlowski K. (2013) Distinct protein classes in human red cell proteome revealed by similarity of phylogenetic profiles. PLoS One, 8, e54471. 23349899 10.1371/journal.pone.0054471 PMC3549994

[25] Bjorling E. Uhlen M. (2008) Antibodypedia, a portal for sharing antibody and antigen validation data. Mol. Cell. Proteomics, 7, 2028–2037. 18667413 10.1074/mcp.M800264-MCP200

[26] Kasza I. Varady G. Andrikovics H. (2012) Expression levels of the ABCG2 multidrug transporter in human erythrocytes correspond to pharmacologically relevant genetic variations. PLoS One, 7, e48423. 23166586 10.1371/journal.pone.0048423 PMC3499528

[27] Trakarnsanga K. Wilson M.C. Griffiths R.E. (2014) Qualitative and quantitative comparison of the proteome of erythroid cells differentiated from human iPSCs and adult erythroid cells by multiplex TMT labelling and nanoLC-MS/MS. PLoS One, 9, e100874. 25019302 10.1371/journal.pone.0100874 PMC4096399

