# Supplementary material for: Inconsistencies in the red blood cell membrane proteome analysis: generation of a database for research and diagnostic applications
Source: Database (Oxford). 2015 Jun 13;2015:bav056. doi: 10.1093/database/bav056 (PMC4480073; doi:10.1093/database/bav056)
Supplement: Supplementary Data [file supp_2015_bav056_index.html]

Inconsistencies in the red blood cell membrane proteome analysis: generation of a database for research and diagnostic applications — Supplementary Data 

# Inconsistencies in the red blood cell membrane proteome analysis: generation of a database for research and diagnostic applications

## Supplementary Data

files

- Supplementary Data - zip file
