# Supplementary material for: Inconsistencies in the red blood cell membrane proteome analysis: generation of a database for research and diagnostic applications
Source: Database (Oxford). 2015 Jun 13;2015:bav056. doi: 10.1093/database/bav056 (PMC4480073; doi:10.1093/database/bav056)
Supplement: Supplementary Data [file supp_bav056_suppl_data.zip › New Microsoft Office Word Document.docx]

**Supporting Information Captions**

**Figure S1. Comparison between the “exclusive human blood peripheral proteome” (Haudek *et al.*, 2009) and the erythrocyte membrane proteome as determined in the present study.** Additionally, 77 proteins are shared between more than one blood constituents proteome**.**

**Figure S2. RBCC (Red Blood Cell Collection) search options.** Complex full text queries with examples and syntax at the bottom of the page can be entered. By default the search is constrained to membrane proteins. Depending on the objective of the query, the confidence of the presence of the matches can be set to low (only semi-automatic identification from reviews), medium (present in either hRBCD or BSc_CH), and high (present in both hRBCD and BSc_CH; a blood group or CD marker; experimentally tested in the Sarkadi-lab). The sources to use in the query can be also selected.

**Figure S3.** **The protein entry page in RBCC.** List of sources, which have identified the presence of the protein in RBC, alternative names, genetic variants and links to other databases are shown in this page. A link to Antibodypedia aids to find an antibody for diagnostics development. Importantly, a pre-built PubMed query is also available to search for papers with all the alternative gene and protein names limiting the search containing alternatives of RBC (e.g. erythrocyte). If the user is logged in, he or she can leave comments for the entry in a box at the bottom of the page.

**Table S1. Proteins and peptide fragments identified in RBC ghosts by MS.**

**Table S2. Hallmark proteins of different blood cell types present in our samples.**

**Table S3. Erythrocyte membrane antigens listed in the CD system** (http://[www.hcdm.or](http://www.hcdm.org)g).

**Table S4. Erythrocyte membrane proteins listed in the human blood group database (BGMUT)** at the NCBI web site ([http://www.ncbi.nlm.nih.gov/ projects/gv/rbc/xslcgi.fcgi?cmd=bgmut/home](http://www.ncbi.nlm.nih.gov/%20projects/gv/rbc/xslcgi.fcgi?cmd=bgmut/home)).
