# Supplementary material for: Inconsistencies in the red blood cell membrane proteome analysis: generation of a database for research and diagnostic applications
Source: Database (Oxford). 2015 Jun 13;2015:bav056. doi: 10.1093/database/bav056 (PMC4480073; doi:10.1093/database/bav056)
Supplement: Supplementary Data [file supp_bav056_suppl_data.zip › rbcc_supportinginfo.pdf]

Supporting Information to

Hegedűs *et al.* : **Inconsistencies in the red blood cell membrane proteome analysis: generation of a database for research and diagnostic applications**

Figure S1. Comparison between the "exclusive human blood peripheral proteome" (Haudek *et al.*, 2009) and the erythrocyte membrane proteome as determined in the present study. Additionally, 77 proteins are shared between more than one blood constituents proteome**.**

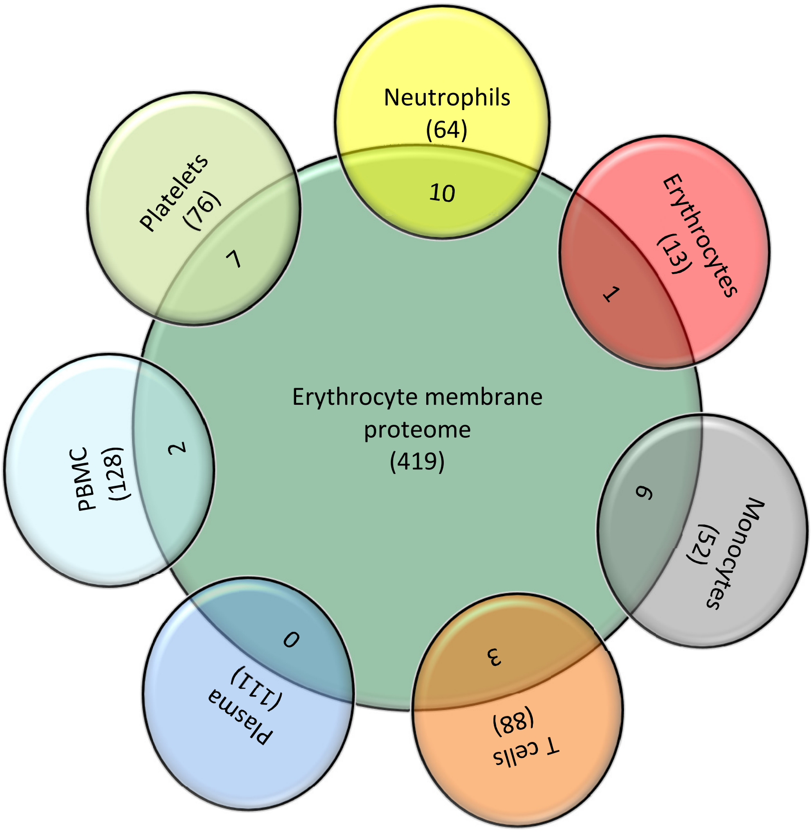

Figure S2. RBCC (Red Blood Cell Collection) search options. Complex full text queries with examples and syntax at the bottom of the page can be entered. By default the search is constrained to membrane proteins. Depending on the objective of the query, the confidence of the presence of the matches can be set to low (only semi-automatic identification from reviews), medium (present in either hRBCD or BSc_CH), and high (present in both hRBCD and BSc_CH; a blood group or CD marker; experimentally tested in the Sarkadi-lab). The sources to use in the query can be also selected.

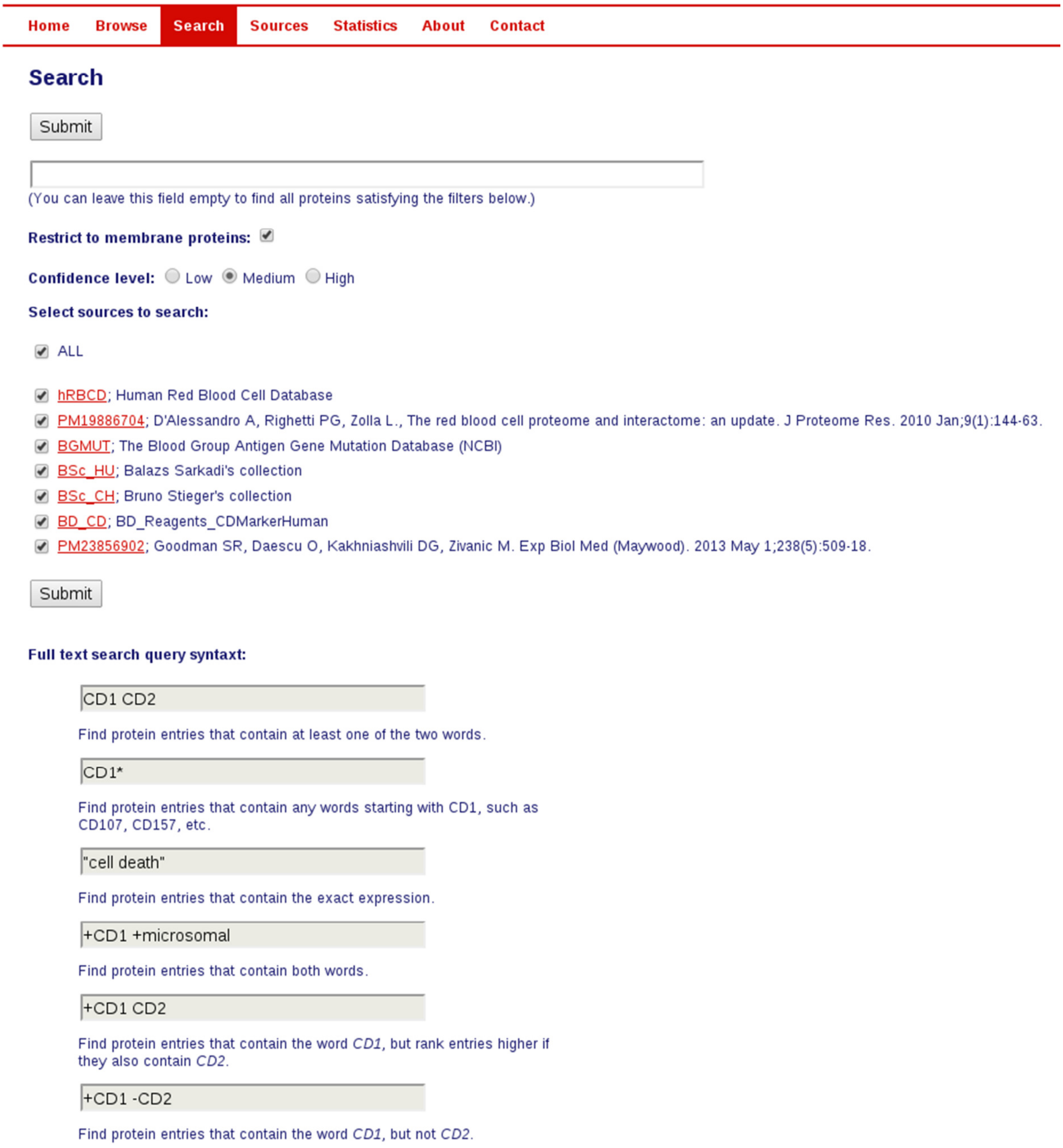

Figure S3. The protein entry page in RBCC. List of sources, which have identified the presence of the protein in RBC, alternative names, genetic variants and links to other databases are shown in this page. A link to Antibodypedia aids to find an antibody for diagnostics development. Importantly, a pre-built PubMed query is also available to search for papers with all the alternative gene and protein names limiting the search containing alternatives of RBC (e.g. erythrocyte). If the user is logged in, he or she can leave comments for the entry in a box at the bottom of the page.

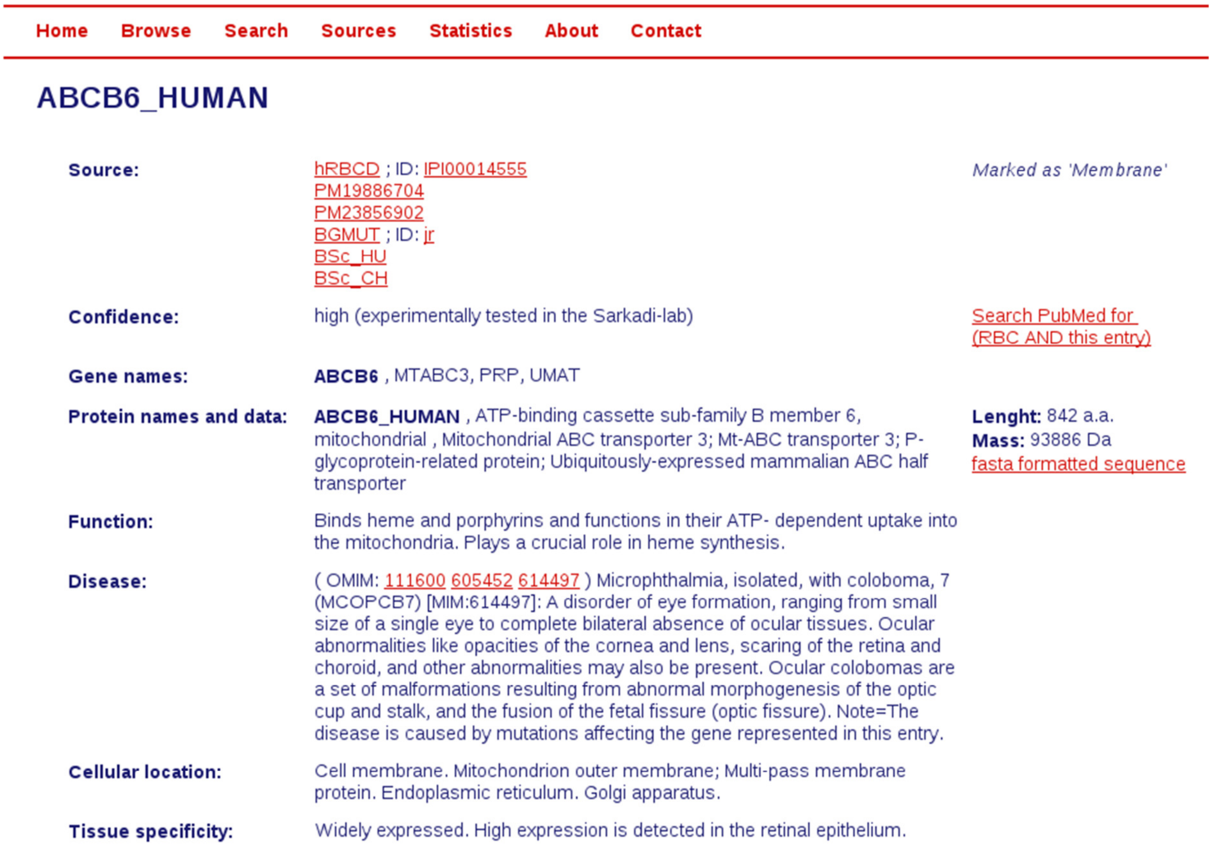

Table S1. Proteins and peptide fragments identified in RBC ghosts by MS.

Table S2. Hallmark proteins of different blood cell types present in our samples.

These supplemental tables can be downloaded from
http://rbcc.hegelab.org/Supplemental_Tables_1_2.xlsx

Table S3. Erythrocyte membrane antigens listed in the CD system (http://www.hcdm.org).

RBC membrane protein	alternative names	listed in RBCC databases
CD109	CPAMD7, CD109 antigen, 150 kDa TGF-beta-1-binding protein, C3 and PZP-like alpha-2-macroglobulin domain-containing protein 7, Platelet-specific Gov antigen, p180, r150, CD109	BD_CD
CSF1R	FMS, Macrophage colony-stimulating factor 1 receptor, CSF-1 receptor, CSF-1-R, CSF-1R, M-CSF-R, 2.7.10.1, Proto-oncogene c-Fms, CD115	BD_CD
CSF2RA	CSF2R, CSF2RY, Granulocyte-macrophage colony-stimulating factor receptor subunit alpha, GM-CSF-R-alpha, GMCSFR-alpha, GMR-alpha, CDw116, CD116	BD_CD
CSF2RB	IL3RB, IL5RB, Cytokine receptor common subunit beta, CDw131, GM-CSF/IL-3/IL-5 receptor common beta subunit, CD131	BD_CD
CSF3R	GCSFR, Granulocyte colony-stimulating factor receptor, G-CSF receptor, G-CSF-R, CD114	BD_CD
ENG	END, Endoglin, CD105	PM19886704, PM23856902, BD_CD
IFNGR1	Interferon gamma receptor 1, IFN-gamma receptor 1, IFN-gamma-R1, CDw119, CD119	BD_CD
IL1R1	IL1R, IL1RA, IL1RT1, Interleukin-1 receptor type 1, IL-1R-1, IL-1RT-1, IL-1RT1, CD121 antigen-like family member A, Interleukin-1 receptor alpha, IL-1R-alpha, Interleukin-1 receptor type I, p80, CD121a, Interleukin-1 receptor type 1, membrane form, mIL-1R1, mIL-1RI, Interleukin-1 receptor type 1, soluble form, sIL-1R1, sIL-1RI	BD_CD
IL1R2	IL1RB, Interleukin-1 receptor type 2, IL-1R-2, IL-1RT-2, IL-1RT2, CD121 antigen-like family member B, CDw121b, IL-1 type II receptor, Interleukin-1 receptor beta, IL-1R-beta, Interleukin-1 receptor type II, CD121b, Interleukin-1 receptor type 2, membrane form, mIL-1R2, mIL-1RII, Interleukin-1 receptor type 2, soluble form, sIL-1R2, sIL-1RII	BD_CD

IL2RB	Interleukin-2 receptor subunit beta, IL-2 receptor subunit beta, IL-2R subunit beta, IL-2RB, High affinity IL-2 receptor subunit beta, p70-75, p75, CD122	BD_CD
IL2RG	Cytokine receptor common subunit gamma, Interleukin-2 receptor subunit gamma, IL-2 receptor subunit gamma, IL-2R subunit gamma, IL-2RG, gammaC, p64, CD132	BD_CD
IL3RA	IL3R, Interleukin-3 receptor subunit alpha, IL-3 receptor subunit alpha, IL-3R subunit alpha, IL-3R-alpha, IL-3RA, CD123	BD_CD
IL4R	IL4RA, 582J2.1, Interleukin-4 receptor subunit alpha, IL-4 receptor subunit alpha, IL-4R subunit alpha, IL-4R-alpha, IL-4RA, CD124, Soluble interleukin-4 receptor subunit alpha, Soluble IL-4 receptor subunit alpha, Soluble IL-4R-alpha, sIL4Ralpha/prot, IL-4-binding protein, IL4-BP	BD_CD
IL5RA	IL5R, Interleukin-5 receptor subunit alpha, IL-5 receptor subunit alpha, IL-5R subunit alpha, IL-5R-alpha, IL-5RA, CDw125, CD125	BD_CD
IL6R	Interleukin-6 receptor subunit alpha, IL-6 receptor subunit alpha, IL-6R subunit alpha, IL-6R-alpha, IL-6RA, IL-6R 1, Membrane glycoprotein 80, gp80, CD126	BD_CD
IL6ST	Interleukin-6 receptor subunit beta, IL-6 receptor subunit beta, IL-6R subunit beta, IL-6R-beta, IL-6RB, CDw130, Interleukin-6 signal transducer, Membrane glycoprotein 130, gp130, Oncostatin-M receptor subunit alpha, CD130	BD_CD
IL7R	Interleukin-7 receptor subunit alpha, IL-7 receptor subunit alpha, IL-7R subunit alpha, IL-7R-alpha, IL-7RA, CDw127, CD127	BD_CD
IL9R	Interleukin-9 receptor, IL-9 receptor, IL-9R, CD129	BD_CD
KIT	SCFR, Mast/stem cell growth factor receptor Kit, SCFR, 2.7.10.1, Piebald trait protein, PBT, Proto-oncogene c-Kit, Tyrosine-protein kinase Kit, p145 c-kit, v-kit Hardy-Zuckerman 4 feline sarcoma viral oncogene homolog, CD117	BD_CD
LAMP1	Lysosome-associated membrane glycoprotein 1, LAMP-1, Lysosome-associated membrane protein 1, CD107 antigen-like family member A, CD107a	PM23856902, BD_CD, BSc_CH
LAMP2	Lysosome-associated membrane glycoprotein 2, LAMP-2, Lysosome-associated membrane protein 2, CD107 antigen-like family member B, CD107b	BD_CD
LIFR	Leukemia inhibitory factor receptor, LIF receptor, LIF-R, CD118	PM23856902,

		BD_CD
MPL	TPOR, Thrombopoietin receptor, TPO-R, Myeloproliferative leukemia protein, Proto-oncogene c-Mpl, CD110	BD_CD
PVRL1	HVEC, PRR1, Poliovirus receptor-related protein 1, Herpes virus entry mediator C, Herpesvirus entry mediator C, HveC, Herpesvirus Ig-like receptor, HIgR, Nectin-1, CD111	PM23856902, BD_CD, BSc_CH
PVRL2	HVEB, PRR2, Poliovirus receptor-related protein 2, Herpes virus entry mediator B, Herpesvirus entry mediator B, HveB, Nectin-2, CD112	BD_CD
PVRL3	PRR3, Poliovirus receptor-related protein 3, CDw113, Nectin-3, CD113	BD_CD
SEMA7A	CD108, SEMAL, Semaphorin-7A, CDw108, JMH blood group antigen, John-Milton-Hargen human blood group Ag, Semaphorin-K1, Sema K1, Semaphorin-L, Sema L, CD108	hRBCD, PM19886704, BGMUT, BSc_CH, BD_CD, PM23856902
TNFRSF1A	TNFAR, TNFR1, Tumor necrosis factor receptor superfamily member 1A, Tumor necrosis factor receptor 1, TNF-R1, Tumor necrosis factor receptor type I, TNF-RI, TNFR-I, p55, p60, CD120a, Tumor necrosis factor receptor superfamily member 1A, membrane form, Tumor necrosis factor-binding protein 1, TBPI	BD_CD
TNFRSF1B	TNFBR, TNFR2, Tumor necrosis factor receptor superfamily member 1B, Tumor necrosis factor receptor 2, TNF-R2, Tumor necrosis factor receptor type II, TNF-RII, TNFR-II, p75, p80 TNF-alpha receptor, CD120b, Etanercept, Tumor necrosis factor receptor superfamily member 1b, membrane form, Tumor necrosis factor-binding protein 2, TBP-2, TBPII	BD_CD
VCAM1	L1CAM, Vascular cell adhesion protein 1, V-CAM 1, VCAM-1, INCAM-100, CD106	BD_CD

Table S4. Erythrocyte membrane proteins listed in the human blood group database (BGMUT) at the NCBI web site (http://www.ncbi.nlm.nih.gov/projects/gv/rbc/xslcgi.fcgi?cmd=bgmut/home).

RBC membrane protein	alternative names	listed in RBCC databases
A4GALT	A14GALT, A4GALT1, Lactosylceramide 4-alpha-galactosyltransferase, 2.4.1.228, Alpha-1, 4-N-acetylglucosaminyltransferase, Alpha-1, 4-galactosyltransferase, Alpha4Gal-T1, CD77 synthase, Globotriaosylceramide synthase, Gb3 synthase, P1/Pk synthase, UDP-galactose:beta-D-galactosyl-beta1-R 4-alpha-D-galactosyltransferase	BGMUT
ABCB6	MTABC3, PRP, UMAT, ATP-binding cassette sub-family B member 6, mitochondrial, Mitochondrial ABC transporter 3, Mt-ABC transporter 3, P-glycoprotein-related protein, Ubiquitously-expressed mammalian ABC half transporter	hRBCD, PM19886704, BGMUT, BSc_HU, BSc_CH, PM23856902
ABCG2	ABCP, BCRP, BCRP1, MXR, ATP-binding cassette sub-family G member 2, Breast cancer resistance protein, CDw338, Mitoxantrone resistance-associated protein, Placenta-specific ATP-binding cassette transporter, Urate exporter, CD338	hRBCD, PM19886704, BGMUT, BSc_HU, BSc_CH, PM23856902
ABO	Histo-blood group ABO system transferase, Fucosylglycoprotein 3-alpha-galactosyltransferase, Fucosylglycoprotein alpha-N-acetylgalactosaminyltransferase, Glycoprotein-fucosylgalactoside alpha-N-acetylgalactosaminyltransferase, 2.4.1.40, Glycoprotein-fucosylgalactoside alpha-galactosyltransferase, 2.4.1.37, Histo-blood group A transferase, A transferase, Histo-blood group B transferase, B transferase, NAGAT, Fucosylglycoprotein alpha-N-acetylgalactosaminyltransferase soluble form	BGMUT
ACHE	Acetylcholinesterase, AChE, 3.1.1.7	hRBCD,

		PM19886704, BGMUT, BSc_CH, PM23856902
AQP1	CHIP28, Aquaporin-1, AQP-1, Aquaporin-CHIP, Urine water channel, Water channel protein for red blood cells and kidney proximal tubule	hRBCD, PM19886704, BGMUT, BSc_CH, PM23856902
AQP3	Aquaporin-3, AQP-3, Aquaglyceroporin-3	BGMUT, BSc_CH
ART4	DO, DOK1, Ecto-ADP-ribosyltransferase 4, 2.4.2.31, ADP-ribosyltransferase C2 and C3 toxin-like 4, ARTC4, Dombrock blood group carrier molecule, Mono(ADP-ribosyl)transferase 4, NAD(P)(+)--arginine ADP-ribosyltransferase 4, CD297	hRBCD, PM23856902, BGMUT, BSc_CH
B3GALNT1	B3GALT3, UNQ531/PRO1074, UDP-GalNAc:beta-1, 3-N-acetylgalactosaminyltransferase 1, Beta-1, 3-GalNAc-T1, 2.4.1.79, Beta-1, 3-galactosyltransferase 3, Beta-1, 3-GalTase 3, Beta3Gal-T3, Beta3GalT3, b3Gal-T3, Beta-3-Gx-T3, Galactosylgalactosylglucosylceramide beta-D-acetyl-galactosaminyltransferase, Globoside synthase, UDP-N-acetylgalactosamine:globotriaosylceramide beta-1, 3-N-acetylgalactosaminyltransferase	BGMUT
BCAM	LU, MSK19, Basal cell adhesion molecule, Auberger B antigen, B-CAM cell surface glycoprotein, F8/G253 antigen, Lutheran antigen, Lutheran blood group glycoprotein, CD239	hRBCD, PM19886704, BGMUT, BSc_CH, PM23856902
BSG	UNQ6505/PRO21383, Basigin, 5F7, Collagenase stimulatory factor, Extracellular matrix metalloproteinase inducer, EMMPRIN, Leukocyte activation antigen M6, OK blood group antigen, Tumor cell-derived collagenase stimulatory factor, TCSF, CD147	hRBCD, PM19886704, PM23856902, BGMUT
C1GALT1	Glycoprotein-N-acetylgalactosamine 3-beta-galactosyltransferase 1, 2.4.1.122, B3Gal-T8, Core 1 O-glycan T-synthase, Core 1 UDP-galactose:N-acetylgalactosamine-alpha-R beta 1, 3-galactosyltransferase 1, Beta-1, 3-galactosyltransferase, Core 1	BGMUT

	beta1, 3-galactosyltransferase 1, C1GalT1, Core 1 beta3-Gal-T1	
C1GALT1C1	COSMC, HSPC067, MSTP143, UNQ273/PRO310, C1GALT1-specific chaperone 1, C38H2-like protein 1, C38H2-L1, Core 1 beta1, 3-galactosyltransferase 2, C1Gal-T2, C1GalT2, Core 1 beta3-Gal-T2, Core 1 beta3-galactosyltransferase-specific molecular chaperone	BGMUT
C4A	CO4, CPAMD2, Complement C4-A, Acidic complement C4, C3 and PZP-like alpha-2-macroglobulin domain-containing protein 2, Complement C4 beta chain, Complement C4-A alpha chain, C4a anaphylatoxin, C4b-A, C4d-A, Complement C4 gamma chain	PM23856902, BGMUT
C4B	CO4, CPAMD3, Complement C4-B, Basic complement C4, C3 and PZP-like alpha-2-macroglobulin domain-containing protein 3, Complement C4 beta chain, Complement C4-B alpha chain, C4a anaphylatoxin, C4b-B, C4d-B, Complement C4 gamma chain	hRBCD, PM19886704, BGMUT, BSc_CH, PM23856902
CD151	TSPAN24, CD151 antigen, GP27, Membrane glycoprotein SFA-1, Platelet-endothelial tetraspan antigen 3, PETA-3, Tetraspanin-24, Tspan-24, CD151	BGMUT
CD44	LHR, MDU2, MDU3, MIC4, CD44 antigen, CDw44, Epican, Extracellular matrix receptor III, ECMR-III, GP90 lymphocyte homing/adhesion receptor, HUTCH-I, Heparan sulfate proteoglycan, Hermes antigen, Hyaluronate receptor, Phagocytic glycoprotein 1, PGP-1, Phagocytic glycoprotein I, PGP-I, CD44	hRBCD, PM19886704, BGMUT, BSc_CH, PM23856902
CD55	CR, DAF, Complement decay-accelerating factor, CD55	hRBCD, PM19886704, BGMUT, BSc_HU, BSc_CH, PM23856902
CD99	MIC2, MIC2X, MIC2Y, CD99 antigen, 12E7, E2 antigen, Protein MIC2, T-cell surface glycoprotein E2, CD99	hRBCD, PM19886704, BGMUT, BSc_CH, PM23856902
CR1	C3BR, Complement receptor type 1, C3b/C4b receptor, CD35	hRBCD, PM19886704,

		BGMUT, BSc_CH, PM23856902
DARC	ACKR1, FY, GPD, Duffy antigen/chemokine receptor, Atypical chemokine receptor 1, Fy glycoprotein, GpFy, Glycoprotein D, Plasmodium vivax receptor, CD234	hRBCD, PM19886704, BGMUT, BSc_CH, PM23856902
ERMAP	RD, SC, Erythroid membrane-associated protein, hERMAP, Radin blood group antigen, Scianna blood group antigen	hRBCD, PM19886704, BGMUT, BSc_CH
FUT1	H, HSC, Galactoside 2-alpha-L-fucosyltransferase 1, 2.4.1.69, Alpha(1, 2)FT 1, Blood group H alpha 2-fucosyltransferase, Fucosyltransferase 1, GDP-L-fucose:beta-D-galactoside 2-alpha-L-fucosyltransferase 1	BGMUT
FUT2	SEC2, Galactoside 2-alpha-L-fucosyltransferase 2, 2.4.1.69, Alpha(1, 2)FT 2, Fucosyltransferase 2, GDP-L-fucose:beta-D-galactoside 2-alpha-L-fucosyltransferase 2, SE2, Secretor blood group alpha-2-fucosyltransferase, Secretor factor, Se	BGMUT
FUT3	FT3B, LE, Galactoside 3(4)-L-fucosyltransferase, 2.4.1.65, Blood group Lewis alpha-4-fucosyltransferase, Lewis FT, Fucosyltransferase 3, Fucosyltransferase III, FucT-III	BGMUT
FUT6	FCT3A, Alpha-(1, 3)-fucosyltransferase 6, 2.4.1.65, Fucosyltransferase 6, Fucosyltransferase VI, Fuc-TVI, FucT-VI, Galactoside 3-L-fucosyltransferase	BGMUT
FUT7	Alpha-(1, 3)-fucosyltransferase 7, 2.4.1.-, Fucosyltransferase 7, Fucosyltransferase VII, Fuc-TVII, FucT-VII, Galactoside 3-L-fucosyltransferase, Selectin ligand synthase	PM23856902, BGMUT
GBGT1	UNQ2513/PRO6002, Globoside alpha-1, 3-N-acetylgalactosaminyltransferase 1, 2.4.1.-, Forssman glycolipid synthase-like protein	BGMUT
GCNT2	GCNT5, II, NACGT1, N-acetyllactosaminide beta-1, 6-N-acetylglucosaminyl-transferase, isoform A, N-acetylglucosaminyltransferase, 2.4.1.150, I-branching enzyme, IGNT	BGMUT
GYPA	GPA, Glycophorin-A, MN sialoglycoprotein, PAS-2,	hRBCD,

	Sialoglycoprotein alpha, CD235a	PM19886704, BGMUT, BSc_HU, BSc_CH, PM23856902
GYPB	GPB, Glycophorin-B, PAS-3, SS-active sialoglycoprotein, Sialoglycoprotein delta, CD235b	PM23856902, BGMUT
GYPC	GLPC, GPC, Glycophorin-C, Glycoconnectin, Glycophorin-D, GPD, Glycoprotein beta, PAS-2', Sialoglycoprotein D, CD236	hRBCD, PM19886704, BGMUT, BSc_CH, PM23856902
GYPE	GPE, Glycophorin-E	BGMUT
ICAM4	LW, Intercellular adhesion molecule 4, ICAM-4, Landsteiner-Wiener blood group glycoprotein, LW blood group protein, CD242	hRBCD, PM19886704, BGMUT, BSc_CH, PM23856902
KEL	Kell blood group glycoprotein, 3.4.24.-, CD238	hRBCD, PM19886704, BGMUT, BSc_CH, PM23856902
RHAG	RH50, Ammonium transporter Rh type A, Erythrocyte membrane glycoprotein Rh50, Erythrocyte plasma membrane 50 kDa glycoprotein, Rh50A, Rhesus blood group family type A glycoprotein, Rh family type A glycoprotein, Rh type A glycoprotein, Rhesus blood group-associated ammonia channel, Rhesus blood group-associated glycoprotein, CD241	hRBCD, PM19886704, BGMUT, BSc_CH, PM23856902
RHCE	RHC, RHE, Blood group Rh(CE) polypeptide, Rh polypeptide 1, RhPI, Rh30A, RhIXB, Rhesus C/E antigens, CD240CE	hRBCD, PM19886704, BGMUT, BSc_CH, PM23856902
RHD	Blood group Rh(D) polypeptide, RHXIII, Rh polypeptide 2, RhPII, Rhesus D antigen, CD240D	PM19886704, PM23856902,

		BGMUT, BSc_CH
SEMA7A	CD108, SEMAL, Semaphorin-7A, CDw108, JMH blood group antigen, John-Milton-Hargen human blood group Ag, Semaphorin-K1, Sema K1, Semaphorin-L, Sema L, CD108	hRBCD, PM19886704, BGMUT, BSc_CH, BD_CD, PM23856902
SLC14A1	HUT11, JK, RACH1, UT1, UTE, Urea transporter 1, Solute carrier family 14 member 1, Urea transporter, erythrocyte	hRBCD, PM19886704, BGMUT, BSc_HU, BSc_CH, PM23856902
SLC4A1	AE1, DI, EPB3, Band 3 anion transport protein, Anion exchange protein 1, AE 1, Anion exchanger 1, Solute carrier family 4 member 1, CD233	hRBCD, PM19886704, BGMUT, BSc_CH, PM23856902
SMIM1	Small integral membrane protein 1, Vel blood group antigen	PM23856902, BGMUT, BSc_CH
XG	PBDX, Glycoprotein Xg, Protein PBDX	BGMUT
XK	XKR1, XRG1, Membrane transport protein XK, Kell complex 37 kDa component, Kx antigen, XK-related protein 1	hRBCD, PM19886704, BGMUT, BSc_CH, PM23856902
